# Restoring the Bite with Gold Crowns

**Published:** 1893-04

**Authors:** H. W. Arthur


ARTICLE V.

### RESTORING THE BITE WITH GOLD CROWNS.

BY DR. H. W. ARTHUR.

Great stress is constantly laid on the careful fitting of the band; this is altogether proper, but *any* bite will do, judging from observation, so that it does not interfere with the occlusion of the other teeth. Apart from the importance to the patient of having the bite *restored*, which is accomplished by this method, the force is in the right direction, and there is no leverage exerted which has a tendency to loosen the crown and, it may be, expand a well-fitted band.

Having the band fitted and not interfering with the occlusion of the teeth, the bite is taken, preferably with modeling composition, which can be hardened with cold water while in the mouth, so that the band can be replaced exactly, should it not draw out with the impression. When in the articulator it should be an exact working bite. Place sufficient wax in the band, now in the articulator and close; this will give the bite in the wax; trim to the band, and give such artistic shape as desired, being careful to leave the bite proper intact. Remove from the articulator (this by the way, can be done more readily if the band be filled with wax, leaving only a slight rim, before placing in the articulator). Take rubber hose about an inch in length and the same in diameter; obstruct the lower half, mix and fill the upper half with Teague's Impression Compound, while in a creamy state, and imbed the wax bite till it slightly covers the band. If the depression in the bite is deep, it may be well to place a little of the compound in it first, to avoid having an air space. When set, the hand and wax bite is removed, and the compound thoroughly dried. This can be quickly done by putting it into a melting-ladle. A change of shade all over will indicate that the moisture has been driven off. Using the rubber ring, dies are made of Melotte's fusible metal. These should be exact and well defined.

A piece of thin, pure gold plate, of sufficient size, is pressed by the fingers, and burnished on the male die, the corners clipped, ends folded in and the sides over, to prevent drawing the gold over the cusps. Strike up once on the dies, and trim off the surplus with light scissors. Place all the parts in the articulator, gold cap bite over the wax bite, close the articulator with slight pressure, burnish the cap down around the band and make sure that it is exactly in place. A trifle of flux wax may be placed where the cap and band meet. Invest, allowing the investment to touch the band at one or more points, so that should it be displaced when the wax is being removed from the inside, it may be exactly replaced. Solder and finish.

Any slight error in manipulation can be noted by placing in the articulator, and can be corrected before placing it in the mouth.—*Ohio Journal.*

